# Reconfigurable Multiparameter Biosignal Acquisition SoC for Low Power Wearable Platform

**DOI:** 10.3390/s16122002

**Published:** 2016-11-25

**Authors:** Jongpal Kim, Hyoungho Ko

**Affiliations:** 1 Samsung Electronics Inc., Suwon 16678, Korea; jongpalk@samsung.com; 2 Department of Electronics Engineering, Chungnam National University, Daejeon 34134, Korea

**Keywords:** biosignal acquisition, reconfigurable circuit, analog front-end, electrocardiogram, photoplethysmography, blood glucose concentration, bioimpedance

## Abstract

A low power and low noise reconfigurable analog front-end (AFE) system on a chip (SoC) for biosignal acquisition is presented. The presented AFE can be reconfigured for use in electropotential, bioimpedance, electrochemical, and photoelectrical modes. The advanced healthcare services based on multiparameter physiological biosignals can be easily implemented with these multimodal and highly reconfigurable features of the proposed system. The reconfigurable gain and input referred noise of the core instrumentation amplifier block are 25 dB to 52 dB, and 1 μV_RMS_, respectively. The power consumption of the analog blocks in one readout channel is less than 52 μW. The reconfigurable capability among various modes of applications including electrocardiogram, blood glucose concentration, respiration, and photoplethysmography are shown experimentally.

## 1. Introduction

Wearable sensors have become popular in many application areas including mobile, commercial, medical, entertainment, security, and so on [1]. Recent advances in wearable sensing technologies enable monitoring of many biosignals in an unobtrusive and seamless manner, thus making it possible to provide high-level context-aware healthcare services based on the continuous acquisition of multiparameter physiological biosignals [2].

Several research papers that focus on monitoring key physiological parameters for health care functions are available. For example, an electropotential (EP) mode is used in applications including electrocardiogram (ECG), electromyogram (EMG), and electroencephalogram (EEG) [3,4,5]; impedance (IMP) mode is used for applications including body fat based on body impedance analysis [6,7], respiration rate [8,9], and galvanic skin response [10]; electrochemical (EC) mode is used in applications including blood glucose monitoring [11]; and photoelectric (PE) mode is used in applications including photoplethysmography (PPG) [12,13] and blood oxygen saturation [14,15]. Moreover, recently, as the requirements on integrated multifunctional biosignal acquisition systems are increasing, some research on multiparameter biosignal acquisition in a single system on a chip (SoC) have been reported [16,17,18].

A reconfigurable sensing channel which enables us to change its sensing modality is highly required for low power and small size implementation. An instrumentation amplifier (IA) is one of the key building blocks for implementing the sensing channel. The capacitively-coupled IA (CCIA) and the current-balanced IA (CBIA) are widely used for IA implementation [5]. A reconfigurable sensing channel based on the CCIA was previously reported [18]. In [18], the reconfigurability between the voltage-mode amplification and the transimpedance amplification was implemented using the feedback capacitances in the CCIA.

The CBIA can achieve lower power and higher common mode rejection ratio (CMRR) than the CCIA, because the CBIA is implemented in an open-loop configuration [5]. In the conventional CBIA, however, the reconfigurability is hard to achieve. In this paper, a reconfigurable sensing channel based on the CBIA with multi sensing modalities is presented. Without the reconfigurability, multi analog front-end (AFE) chips for multi biosignal acquisition should be embedded in a wearable system, which is not a preferred situation for a tiny and light form factor. In addition, from the chip maker’s perspective, producing all kinds of chips considering sensing modality and number of sensing channels is inefficient. For a simple product family, it is preferred that the number of sensing channels should be the only criteria for the product family classification. For this reason, our research focused on a reconfigurable sensing channel to be adaptable to various sensing modalities. 

## 2. Design of Reconfigurable AFE

**Figure 1:**
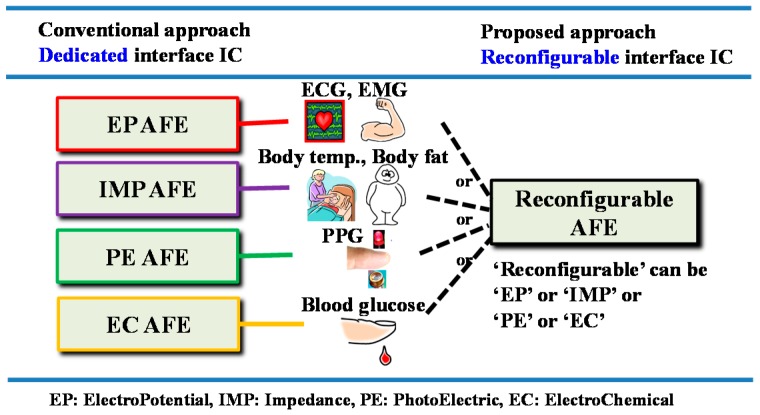
Difference between the conventional and proposed approaches.

**Figure 2:**
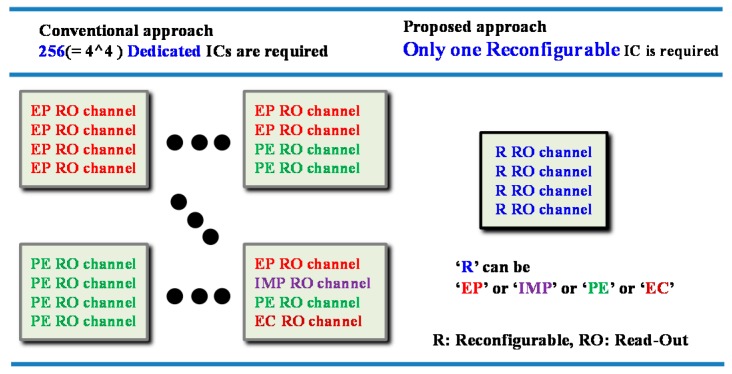
Required number of integrated circuits (ICs) to adapt various applications for four interface channels in the conventional and proposed approaches.

In order to support various biosignals on a single platform, the biosignals were categorized into four sensing modes and we applied reconfiguration capability among the sensing modes into our AFE. A difference between the conventional approach and our proposed AFE approach is shown in Figure 1. In the conventional approach, each sensing mode requires a corresponding AFE circuit. In the proposed approach, however, one AFE can be reconfigured in real time into an EP sensing mode for voltage signals, into an IMP sensing mode for modulated voltage signals, into an EC sensing mode for current signals, or into a PE sensing mode for modulated current signals. For example, if an AFE chip is equipped with four reconfigurable readout channels, then its applicability corresponds to the capabilities of the 256 types of conventional AFE chips as shown in Figure 2. In other words, the ability to reconfigure assigns platform characteristics to the AFE. 

**Figure 3:**
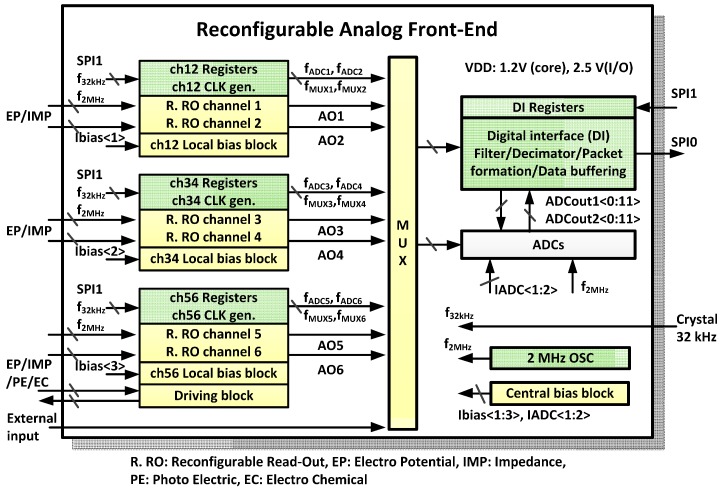
Top overview of the reconfigurable analog front-end (AFE).

An overview of the reconfigurable AFE is shown in Figure 3. Four reconfigurable readout channels between the EP/IMP sensing modes, two reconfigurable readout channels among the EP/IMP/PE/EC sensing modes, analog to digital converters (ADCs), and digital interfaces (DIs) are included. Two reconfigurable readout channels, local bias block, local channel clock generator, and local channel registers are located closely as a group to minimize the number of global control lines and the line length from the bias signal generation block to the readout channel. A central bias block generates and supplies a current reference rather than a voltage reference. The local bias block generates all necessary voltage references using the current reference, which is from the central bias block. All clocks used in readout channels, driving block, ADC multiplexer (MUX), and ADCs are fully programmable. The frequency and repetition pattern of each clock can be defined by control registers, which are fully programmable via the serial peripheral interface (SPI). The repetition pattern can be defined using a 32 or a 256-bit stream register. The user-defined clock pattern can be produced at any desired frequency using a cycle clock. The cycle clock is divided from a base clock of 32 kHz or 2 MHz by user definition. Two 12-bit successive approximation ADCs are integrated and multiplexed to readout channel outputs. A DI block can buffer 512 ADC output data to reduce frequent awakening of main digital processor.

**Figure 4:**
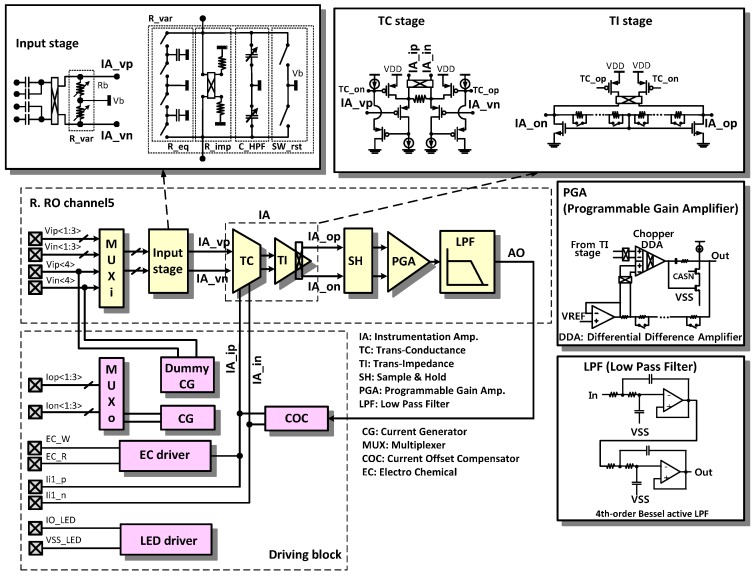
Architecture of the reconfigurable channel.

The reconfigurable readout channel and the driving block are expanded in Figure 4. Depending on the sensing mode, the instrumentation amplifier (IA) should have the capability to measure a voltage input or a current input. In our design, the transconductance (TC) stage can measure voltage inputs at IA_vp and IA_vn and current inputs at IA_ip and IA_in. Conventionally, two types of amplifiers, such as an IA for a voltage measurement and a TC amplifier for a current measurement are required. 

The resistor in the TC stage is 10 kOhm. The resistors in the TI stage are designed with a wide programming range from 25.9 kOhm to 6.6 MOhm with 256 steps using 8-bit registers, to compensate for the large gain variations including the process variations, the electrode impedance, and the human-to-human signal amplitude variations. The programmable gain amplifier (PGA) is implemented using a differential difference amplifier (DDA) with non-inverting amplifier configuration. The 4th order Bessel low pass filter (LPF) is implemented using the Sallen-Key active filter configuration. 

### 2.1. Electropotential (EP) Sensing Mode

**Figure 5:**
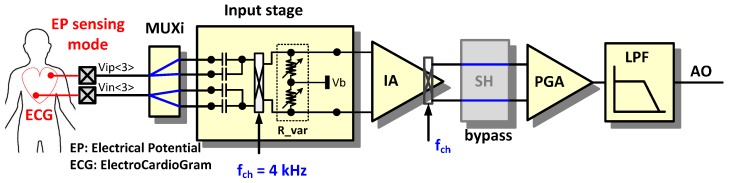
Reconfiguration of the readout channel for EP sensing mode.

In the EP sensing mode, the AFE circuit is configured for voltage sensing [3]. The EP sensing mode can measure ECG, EMG, and EEG signals. Figure 5 shows an example configuration for ECG measurement. Ion-based signals in the body are converted to electron-based input signals through wet or dry electrodes. The input signals are filtered at the input stage with the 0.5 Hz cut-off high-pass filter (HPF) composed of a capacitor and an equivalent resistor. The equivalent resistor is formed by the input chopper’s switch operation on capacitors between the input chopper and the IA. To reduce flicker noise in the IA, a chopper stabilized technique is adopted. The input chopper works as a modulator and the output chopper in the IA works as a demodulator. The sample and hold (SH) block is bypassed. In the programmable gain amplifier (PGA), the output signal of the IA can be amplified additionally. The LPF has an adjustable cut-off frequency between 70 to 500 Hz.

### 2.2. Impedance (IMP) Sensing Mode

In the IMP sensing mode, the AFE circuit is reconfigured for frequency modulated current injection, modulated voltage sensing, and sensed signal demodulation. The IMP sensing mode is applicable to body-composition analysis, respiration detection, body temperature monitoring with a thermistor, and blood pressure measurement with a piezoresistive pressure transducer.

**Figure 6:**
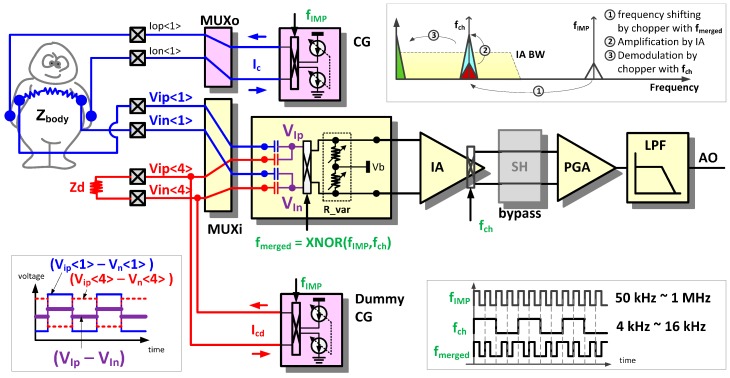
Reconfiguration of the readout channel for impedance (IMP) sensing mode.

Figure 6 shows an example configuration for body impedance measurement. The modulated current (I_c_) from the current generator (CG) is injected into an object, and the induced voltage signals (V_ip<1>_, V_in<1>_) are amplified and demodulated by the IA. In addition to the basic impedance sensing function, two features are added to (i) sense a high-frequency modulated signal using a low bandwidth IA to save on power consumption; and to (ii) trim a DC base impedance using an off-chip dummy impedance to amplify the time-varying component of the sensed impedance.

#### 2.2.1. Intermediate Frequency Shifting Chopper (IFSC) Method Driven by XNOR Merged Clock

To measure the bioelectrical impedance, usually an alternating current of a single frequency in the range of 5 kHz to 1 MHz is injected. To detect the induced high-frequency signal due to such an injection of AC current, an IA with a bandwidth of at least a few MHz is required. To reduce power consumption, however, the IA bandwidth should be restricted to 30 kHz as used in the EP sensing mode. To fulfill these contradictory requirements of sensing over one MHz signal by an IA with a few tens of kHz bandwidth, an intermediate frequency shifting chopper (IFSC) method driven by a XNOR merged clock is proposed.

The frequency domain signal flow in an IFSC method is summarized in the right upper corner of Figure 6 [6]. The induced voltage signals (V_ip<1>_, V_in<1>_) with the high-injection frequency are transferred to an intermediate frequency signal before amplification. The intermediate frequency should be sufficiently low to be within the IA bandwidth and sufficiently high to avoid the flicker noise of the IA as well. The input chopper, driven by the merged clock (f_merged_) in the input stage, performs intermediate frequency shifting. The merged clock (f_merged_) is generated by a XNOR operation of f_IMP_ and f_ch_, as shown in the right lower corner of Figure 6. The two clock sources, f_IMP_ and f_CH_, are programmable, and are static during chopper operations. Thus, the merged clock (f_merged_) is periodic with the periods of the least common multiples of two clock sources. The intermediate frequency signal is amplified and demodulated to the baseband using the output chopper driven by f_ch_.

#### 2.2.2. Base Impedance Trimming Method using Dummy Impedance

In case the relative swing signal is of importance, most of the base portion of the impedance can be cancelled out using dummy impedance and an anti-phase injection current source. By removing the base portion, channel output saturation can be prevented, and the remaining swing signal can be additionally magnified, which enhances the monitoring resolution. Shifting the scope of the impedance measurement to a scope of interest such as 0–600 Ohm, 100–700 Ohm, and 200–800 Ohm is also possible.

In the left lower corner of Figure 6, signal waveforms that occur in the DC impedance trimming method using a dummy impedance can be seen. In addition to the induced signals (V_ip<1>_, V_in<1>_) on the object, the anti-phase voltage signals (V_ip<4>_, V_in<4>_) are induced by the dummy current (I_cd_) from the dummy CG. Then, the input signals at the input chopper are reduced to (V_ip<1>_–V_ip<4>_) and (V_in<1>_–V_in<4>_). By changing a size of Z_d_ or controlling the current magnitude from the dummy CG, the input signal amplitude at the input chopper can be adjusted properly.

### 2.3. Photoelectric (PE) Sensing Mode

In the PE sensing mode, the AFE circuit is reconfigured for frequency modulated current injection, current sensing, and signal restoration. The PE sensing mode is applied to heartbeat rate monitoring, blood vessel stiffness analysis, and pulse transit time (PTT) blood pressure measurement. 

**Figure 7:**
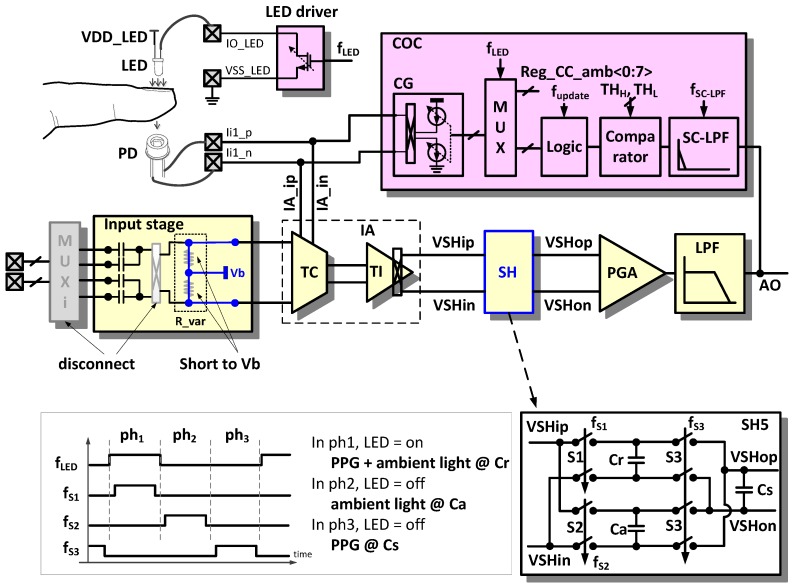
Reconfiguration of the readout channel for photoelectric (PE) sensing mode.

Figure 7 shows an example configuration for PPG measurement [13]. The light-emitting diode (LED) controller controls the amount of light emitted from the LED. The LED is turned on and off for lower power consumption according to the control signal f__LED_. The current signal generated by the photodiode (PD) is injected to the TC stage, converted into a voltage signal in the TI stage, and an ambient light interference is cancelled out in the SH block. A considerable amount of the DC offset from the PD is compensated by the current offset compensator (COC) so that it does not saturate a signal at the ADC input stage.

#### 2.3.1. Ambient Light Cancellation (ALC)

Owing to variations in the ambient light intensity or optical path condition between a light source and a detector, an unwanted AC component is present in a detected signal. Ambient light such as the sun or a fluorescent lamp can be noise because it is unknown and unpredictable.

An ambient light interference in a detected PPG signal can be removed in the SH block. The detailed structure and operation clock diagram are depicted in the lower part of Figure 7. When the LED light turns on (phase ph_1_), the measured signal corresponding to both the LED light and ambient light is sampled at the capacitor Cr, and when the LED light turns off, the measured signal corresponding only to the ambient light is sampled at another capacitor Ca (phase ph_2_). Then, the charge redistribution occurs to cancel the ambient light component by reconnecting the two capacitors in a manner of reversed polarity (phase ph_3_).

#### 2.3.2. Automatic Offset Compensation (AOC)

A DC offset is typically a hundred times larger than a pulse PPG signal in the PD output. The adoption of a wide span of operating voltages is an easy method to prevent signal saturation from the large DC offset; however, this results in inefficient power consumption. Therefore, the DC offset should be removed before the amplification of the detected signal.

The large DC offset current compared with the AC pulse current from the PD is compensated using the COC. The analog output (AO) signal is filtered by the switched capacitor LPF (SC-LPF), which has a cut-off frequency of 0.1 Hz to extract the DC component. The comparator generates the information about whether the input signal causes saturation on the positive supply side, on the negative supply side, or no saturation based on a comparison with the upper and lower threshold values (TH_H_, TH_L_). Depending upon this information, the compensator logic using an up/down counter increases or decreases the compensation current. The current compensation is implemented by updating the value of the frequency f__update_ and is effective when the LED turns on. The COC includes an 8-bit current DAC that compensates up to a 30 μA DC offset.

### 2.4. Electrochemical (EC) Sensing Mode

In the EC sensing mode, the AFE circuit is reconfigured for voltage holding and current sensing. The EC sensing mode is applied in chemical sensing such as blood glucose, cholesterol, and lactic acid sensing.

**Figure 8:**
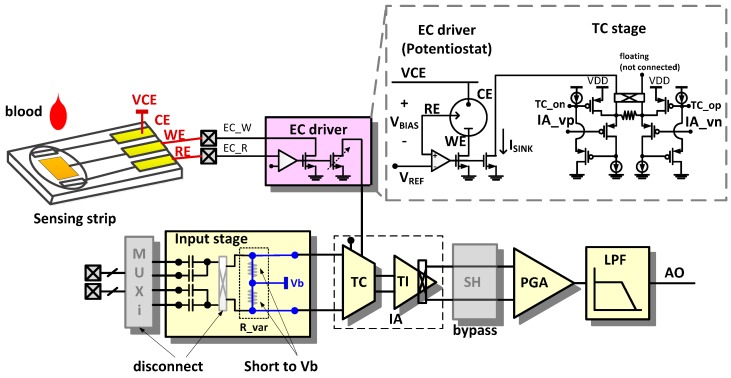
Reconfiguration of the readout channel for electrochemical (EC) sensing mode.

Figure 8 shows a typical configuration for blood glucose measurement. When a 0.95 V and a 0.49 V is applied to the counter electrode (CE) and the reference electrode (RE), respectively, a reaction current through the working electrode (WE) is mirrored to the TC stage. In the EC sensing mode, the AFE is designed to accommodate the sensing current range up to 25 μA for the major commercial sensing strips (One Touch Ultra of J&J, Optium Xceed of Aboott, Accu-Check of Roche, and Contour TS of Bayer).

## 3. Results of the Measurements Using the Reconfigurable AFE

**Figure 9:**
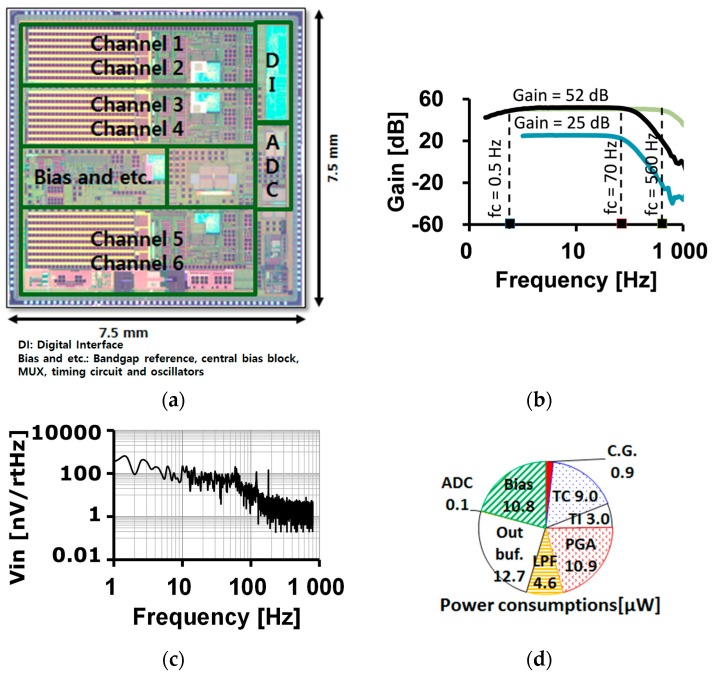
Fabricated AFE and basic characteristics; (**a**) chip photograph; (**b**) readout channel frequency response at PGA gain = 2; (**c**) input referred voltage noise characteristics of the readout channel; (**d**) power consumption breakdown of the analog readout channel.

**Figure 10:**
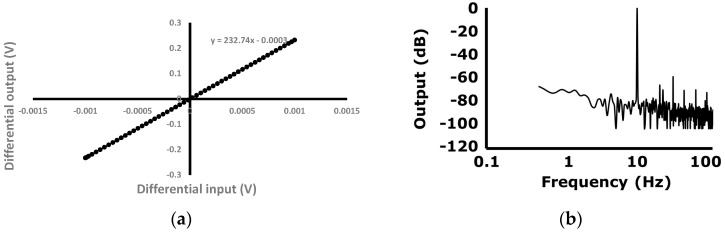
Linearity and total harmonic distortion (THD); (**a**) input-output linearity; (**b**) output spectrum with 1 mV, 10 Hz sinusoidal voltage input.

A photograph of the AFE die, fabricated using a standard 0.13 μm complementary metal oxide semiconductor (CMOS) process, is shown in Figure 9a. In the EP sensing mode, the voltage gain of the readout channel can be adjusted from 25 dB to 52 dB with PGA gain set to 2. The PGA gain also can be adjusted from 1 to 6. The cut-off frequency of the HPF is 0.5 Hz and the adjustable cut-off frequency range of the LPF is measured from 70 Hz to 560 Hz as shown in Figure 9b. The measured input referred voltage noise is shown in Figure 9c. The integrated input referred noise is calculated as 1 μV_RMS_ with a readout channel gain of 180 and a bandwidth of 100 Hz. The power consumption of analog blocks in one readout channel is less than 52 μW as displayed in Figure 9d. The input-output characteristics and the output frequency spectrum with sinusoidal voltage input are shown in Figure 10a,b. The amplitude and the frequency of the input voltage signal is 1 mV and 10 Hz, respectively. The gain is set to be 232.7 v/v. The input-output linearity is measured to be 0.06% FSO (full scale output). The largest harmonic is the third harmonic, which is −58.8 dB lower than the fundamental input frequency. The total harmonic distortion (THD) is 0.131%. 

**Figure 11:**
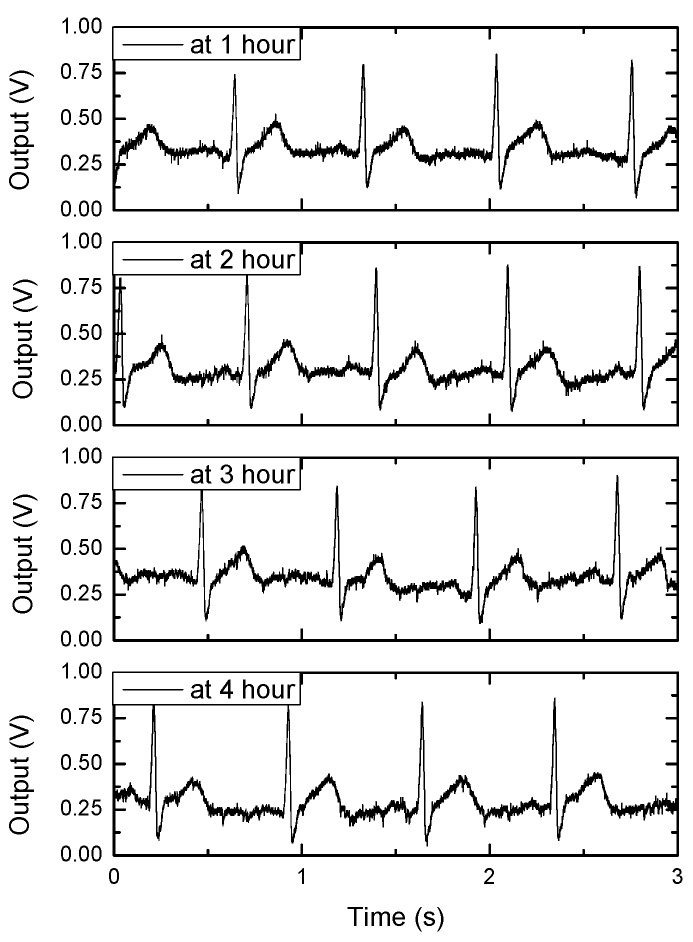
Measured electrocardiogram (ECG) signal in electropotential (EP) mode.

**Figure 12:**
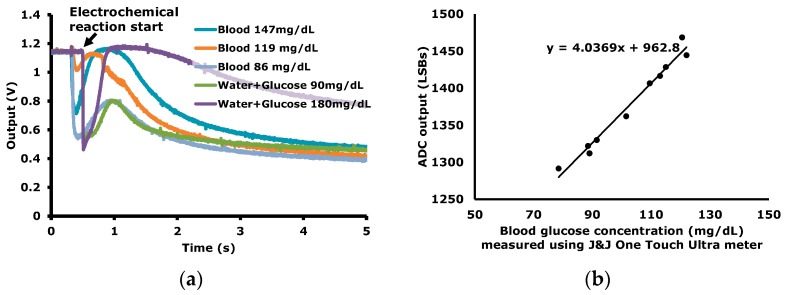
Blood glucose measurement results in EC mode; (**a**) time-domain electro-chemical reaction signal; (**b**) ADC output vs. blood glucose concentration.

The ECG signals are measured and plotted in Figure 11 as an example of the operation in the EP sensing mode. The ECG signals are measured during a 4 h time-period with wet Ag/AgCl electrodes. In the EC sensing mode, the time-domain blood glucose reactions are measured as shown in Figure 12a. For a transducer to measure blood glucose, a commercial strip (OneTouch Ultra Strip of J&J) is used. The peak value or the value at a specific time is correlated to the blood glucose level. In Figure 12b, the ADC output versus the blood concentration levels, which are measured using the commercial blood glucose concentration meter (OneTouch Ultra meter of J&J), are plotted. 

**Figure 13:**
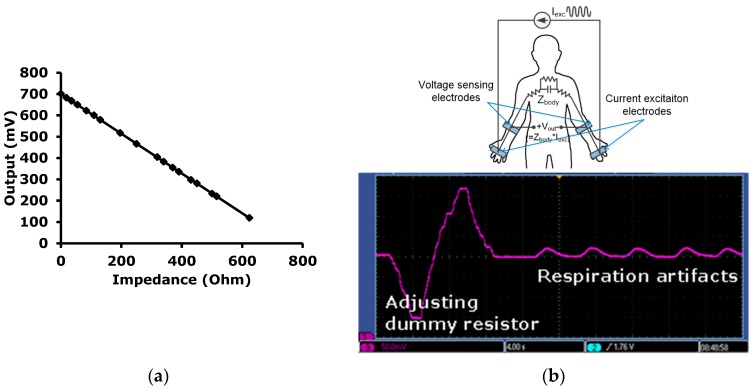
Measurement results in the IMP sensing mode; (**a**) Input-output characteristics; (**b**) DC trimming by changing the value of the dummy resistor and respiration signal.

In the IMP sensing mode, measurement results are shown in Figure 13. The measured impedances with an excitation frequency f_IMP_ of 1.24 MHz using the IFSC method are plotted in Figure 13a. For the measurements shown in Figure 13a, a variable resistor is used. Figure 13b clearly shows the base line movement to the center of a swing range as a result of trimming the dummy resistor during the measurement of a time domain respiration signal. The change of volume of the human body during inhalation and exhalation causes a change in body impedance. 

**Figure 14:**
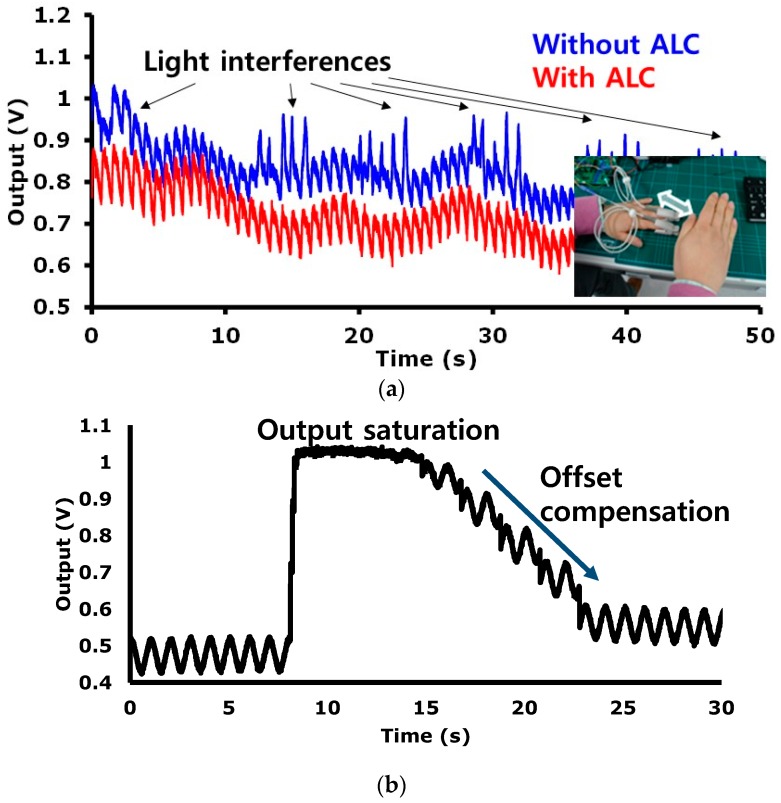
Measurement results in the PE sensing mode; (**a**) photoplethysmography (PPG) signals compared with/without the ambient light cancellation (ALC) function, according to the ambient light noise application; (**b**) Restoration of the saturated signal by the automatic offset compensation (AOC).

In the PE sensing mode, the readout channel has a transimpedance gain of 160 kΩ to 3.5 MΩ. The input referred current noise level is 260 pA_RMS_ in a bandwidth of 100 Hz. To validate the efficacy of the proposed ALC technique, a PPG (probe 1) is equipped on an index finger and an another PPG (probe 2) is equipped on a middle finger as shown in Figure 14a. PPG probe 1 and PPG probe 2 are connected to readout channel 5 and readout channel 6, respectively. Readout channel 5 is set to work with the ALC and readout channel 6 is set to work without the ALC. Owing the variation of the light from a fluorescent lamp, the signal from channel 6 has a peak component that is influenced by the incident ambient light variation, whereas the signal from channel 5 is not influenced by the ambient light variation. Figure 14a shows that the proposed alternate sampling and charge redistribution technique can eliminate an ambient light interference. To evaluate the large DC offset current compensation capability of the proposed technique, a combination sinusoidal current of 1 Hz and a DC are applied as an input. As seen in Figure 14b, due to the large DC current, a monitored signal is saturated initially.

The restoration speed depends on the logic update clock f_update_ shown in Figure 7. In Figure 14b, we used a slow update clock (f_update_ < sub 1 Hz) intentionally to show the compensation process visually. In real implementation, the COC is operated with two phases. In Phase 1, if over-threshold state is detected, a fast logic update clock (ex. 64 Hz) is applied, and then the saturated output signal comes to a near threshold level. In phase 2, if touch of another side threshold voltage is detected, a slow logic update clock (ex. sub Hz) is applied, and then the output signal comes into a region within the thresholds. The DC offset compensation of up to 30 μA is achieved experimentally.

## 4. Conclusions

The low power and low noise AFE SoC with multimodal and highly reconfigurable features is presented. The performance comparisons with the previous multi-modal AFEs are summarized in Table 1. The presented AFE can be reconfigured to the EP, IMP, EC, and PE modes. The reconfigurable gain and input referred noise of the core IA block are 25 dB to 52 dB, and 1 μV_RMS_, respectively. The power consumption of the analog blocks in one readout channel is less than 52 μW. To validate the functionalities of the EP mode and the EC modes, a lead I ECG signal and blood glucose reaction are measured, respectively. For the IMP mode, respiration artifacts are measured using body impedance change. For the PE mode, PPG signals under ambient light interferences are acquired. The multimodal and highly reconfigurable features of the proposed SoC can reduce the required number of chips for implementing multifunctional biosignal acquisitions, thus, the overall reduction of the manufacturing complexity, cost, power consumption, and the system size can be achieved.

## Figures and Tables

**Table 1:** Performance comparisons.

	This Work	[16]	[17]	[18]
Process	0.13 μm	65 nm	0.18 μm	0.35 μm
VDD (V)	1.2	1.2	1.2/1.8	1.8
Power consumption (uW)	52 (readout + bias)	850	73 (ECG readout + bias + ADC)	18.17 (readout + bias)
Input referred noise (uV_rms_) (in 100 Hz BW)	1	N/A	0.82	7.69
Gain (dB)	20~67.5 (8-bit)	42	N/A	0~40 (7-bit)
ADC	12-bit SAR	N/A	15-bit ΔΣ	10-bit SAR
Sensing modalities	ExG/Bio-Z/Glucose/PPG	ExG/Bio-Z/PPG	ECG/Bio-Z/GSR/PPG	V/I/R/C (Glucose/Protein/pH/Temp.)
Reconfigurability	Y	N	N	Y
